# Helminth-Associated Systemic Immune Activation and HIV Co-receptor Expression: Response to Albendazole/Praziquantel Treatment

**DOI:** 10.1371/journal.pntd.0002755

**Published:** 2014-03-27

**Authors:** Mkunde Chachage, Lilli Podola, Petra Clowes, Anthony Nsojo, Asli Bauer, Onesmo Mgaya, Dickens Kowour, Guenter Froeschl, Leonard Maboko, Michael Hoelscher, Elmar Saathoff, Christof Geldmacher

**Affiliations:** 1 National Institute for Medical Research (NIMR)-Mbeya Medical Research Centre, Mbeya, Tanzania; 2 Division of Infectious Diseases and Tropical Medicine, Medical Center of the University of Munich (LMU), Munich, Germany; 3 German Centre for Infection Research (DZIF), Munich, Germany; National Institutes of Health, United States of America

## Abstract

**Background:**

It has been hypothesized that helminth infections increase HIV susceptibility by enhancing systemic immune activation and hence contribute to elevated HIV-1 transmission in sub-Saharan Africa.

**Objective:**

To study systemic immune activation and HIV-1 co-receptor expression in relation to different helminth infections and in response to helminth treatment.

**Methods:**

HIV-negative adults with (n = 189) or without (n = 57) different helminth infections, as diagnosed by Kato-Katz, were enrolled in Mbeya, Tanzania. Blinded to helminth infection status, T cell differentiation (CD45RO, CD27), activation (HLA-DR, CD38) and CCR5 expression was determined at baseline and 3 months after Albendazole/Praziquantel treatment. Plasma cytokine levels were compared using a cytometric bead array.

**Results:**

Trichuris and Ascaris infections were linked to increased frequencies of “activated” CD4 and/or CD8 T cells (p<0.05), whereas Hookworm infection was associated with a trend towards decreased HLA-DR^+^ CD8 T cell frequencies (p = 0.222). In Trichuris infected subjects, there was a linear correlation between HLA-DR^+^ CD4 T cell frequencies and the cytokines IL-1β and IL-10 (p<0.05). Helminth treatment with Albendazole and Praziquantel significantly decreased eosinophilia for *S. mansoni* and Hookworm infections (p<0.005) but not for Trichuris infection and only moderately modulated T cell activation. CCR5 surface density on memory CD4 T cells was increased by 1.2-fold during Trichuris infection (p-value: 0.053) and reduced after treatment (p = 0.003).

**Conclusions:**

Increased expression of T cell activation markers was associated with Trichuris and Ascaris infections with relatively little effect of helminth treatment.

## Author Summary

Helminth infections are common in sub-Saharan Africa where about half of the population may be infected with one or more helminth species. HIV infection is also highly prevalent in this region. Because of the geographic overlap of helminth and HIV infections, it has been hypothesized that helminth infections may increase susceptibility to HIV by increasing systemic immune activation, which has been linked to increased HIV susceptibility. We therefore investigated the profile of T cell activation in individuals infected with different helminth species before and after helminth treatment within the WHIS cohort in Mbeya, Tanzania. Our study shows that systemic T cell activation differs between infections with different helminths. Particularly Trichuris but also Ascaris and *S. mansoni* infections were linked to increased frequencies of activated, HLA-DR^+^ T cells with relatively little effect of helminth treatment. Hookworm infection was associated with a trend towards decreased frequencies of activated, HLA-DR^+^ CD8^+^ T cells. Our study supports the concept that helminth infections, which are linked to systemic immune activation, could potentially also contribute to increased HIV transmission.

## Introduction

In 1995, Bentwich et al. proposed that systemic immune activation associated with chronic helminth infection may be the driving force of HIV transmission in Africa [1] as such infections are common in that environment (reviewed in [2]). Since then, several studies have linked systemic immune activation in African populations to helminth infection [3]–[5]. A series of such studies was conducted in Israel with newly arrived Ethiopian migrants who were characterized by a high prevalence of helminth infections such as Schistosomes, Hookworm, *Ascaris lumbricoides* (Ascaris) or *Trichuris trichiura* (Trichuris). Compared to Ethiopian migrants that had stayed in Israel for longer periods and had received standard anti-helminthic treatment upon arrival, HLA-DR expression on CD4 and CD8 T cells and lymphocyte apoptosis was substantially higher in the new arrivals [3]. Also, peripheral blood mononuclear cells (PBMCs) of these immigrants were highly susceptible to in vitro infection with HIV, which correlated with the state of immune activation [6]. Within a similar study population, the same group also reported higher CCR5 and CXCR4 expression levels in Ethiopians, regardless of the length of their residence in Israel and thus also of the time after anti-helminthic treatment [4]. Contrary to this, a more recent study observed no differences in the T cell immune activation profile of HIV negative subjects between individuals infected with Trichuris and/or Ascaris and non-helminth infected participants, except for a 2-fold increased frequency of CCR5 expression on CD4 T cells in helminth infected subjects [7].

Low systemic immune activation is a correlate of protection against HIV infection [8], [9]. This has been demonstrated in recent human studies which reported that low immune activation in highly HIV-1-exposed but uninfected individuals contributes to their resistance to HIV infection [9], [10]. Koning et al. extensively showed that the blood of high risk but persistently seronegative men from the Amsterdam cohort had lower frequencies of co-expression of HLA-DR and CD38 on CD4 T cells, low proportions of cycling T cells as defined by the expression of Ki67 nuclear antigen and low proportion of memory CD4 T cells expressing CCR5, in comparison to men who were seronegative at the time of analysis but later on became HIV positive [9]. Similarly, Begaud et al. observed significantly lower expression of HLA-DR and CCR5 on CD4 T cells in HIV-1 exposed seronegative heterosexuals from a Central African cohort [10], suggesting a role of CD4 T cell immune activation in HIV susceptibility.

While these studies support a link between systemic T cell activation and HIV susceptibility, it is less clear, whether in populations from endemic areas of sub-Saharan Africa helminth infections in general are associated with systemic T cell activation or whether infections with different helminth species might differ in this regard. In order to elucidate this open question, the present study aimed to investigate systemic T cell activation in relation to infection with different helminth species and to anti-helminthic treatment.

## Materials and Methods

### Ethics statement

This study was approved by the ethics committees of the Tanzanian National Institute for Medical Research, Mbeya Referral Hospital and Munich University and conducted according to the principles expressed in the Declaration of Helsinki. All participants recruited in the study were adults (18–50 years) who provided written informed consent before enrolment into the study.

### Study volunteers

A total of 386 adult study participants from the “Evaluating and Monitoring the Impact of New Interventions” (EMINI) [11] cohort from the Mbeya region in South West Tanzania were enrolled into the prospective Worm_HIV_Interaction_Study (WHIS) cohort based on their helminth and HIV infection status about four months after the EMINI field visit. The initial objective was to only include participants with single helminth infection, however, some participants within the HIV negative group turned out to have multiple helminth infections when re-tested after randomization into the WHIS study (Table 1). 246 HIV negative volunteers were then further stratified according to their helminth-infection status, including 57 helminth negative subjects (Table 1). Blood, urine and stool specimens were collected from each participant once at baseline and once during the follow up at 1–3 months after helminth treatment irrespective of helminth infection status with a single dose of Albendazole (400 mg) and Praziquantel (40 mg/kg). Only subjects without detectable helminth infections after treatment were included in the comparison of pre- and post-treatment time points. Helminth diagnosis was performed as described below. HIV status was determined using HIV 1/2 STAT-PAK, (Chem-bio Diagnostics Systems) and positive results were confirmed using ELISA (Bio-Rad). Discrepancies between HIV 1/2 STAT-PAK and ELISA were resolved by Western Blot (MPD HIV Blot 2.2, MP Biomedicals). 40 ml of venous blood were drawn from each participant using anticoagulant tubes (CPDA, EDTA; BD Vacutainer). Blood samples were processed within less than 6 hours of the blood draw at the MMRC laboratories.

**Table 1:** WHIS study cohort.

	Whole WHIS study cohort	Sub-population for baseline analysis	Sub-population for post-treatment analysis
N	386	246	177
**HIV neg., N (%)**	246 (63.7)	246 (100.0)	177 (100.0)
**Females, N (%)**	235 (60.9)	144 (58.5)	105 (59.3)
**Age, mean (SD)**	34.7 (10.9)	33.6 (11.5)	33.1 (11.7)
**# of Helminth infections**			
none	118 (30.6)	57 (23.2)	42 (23.7)
one	221 (57.3)	159 (64.6)	118 (66.7)
two	40 (10.4)	25 (10.2)	14 (7.9)
three	6 (1.6)	5 (2.0)	3 (1.7)
four	1 (0.3)	0 (0.0)	0 (0.0)

### Diagnosis of helminth species

Fresh stool specimens were used for Kato-Katz diagnosis of geohelminth (Trichuris, Ascaris, Hookworms) and *S. mansoni* infections. Briefly, two Kato-Katz thick smears (41.7 mg each) were prepared from each fresh stool. Kato-Katz slides were microscopically examined for helminth eggs by experienced technicians within one hour (for Hookworm eggs) and within two days (for other helminth eggs) after slide preparation. *S. haematobium* infection was diagnosed by microscopic examination of a filtered urine sample (20 ml) for *S. haematobium* eggs. Helminth infection was defined as the presence of at least one worm egg in the examined samples.

### Quantification of eosinophil counts

An automated complete blood count machine (Beckman Coulter) was used for counting eosinophiles. If eosinophil counts were out of range (>1.0×10^3^/μl), determination was performed using the differential blood count.

### Characterization of maturation and activation markers on CD4 and CD8 T cells in fresh whole blood

Frequencies of activation (HLA-DR, CD38 and CCR5) and maturation (CD27 and CD45R0) markers were determined in fresh, anti-coagulated whole blood at each of the two time points. Blood samples were incubated for 10 minutes with CCR5 PECy7 followed by 30 minutes incubation using the following fluorochrome labeled monoclonal antibodies for cell surface staining (mABs); CD3-Pac Blue (BD), CD4 Per-CP Cy5.5 (eBioscience), CD8 V500 or CD8 Amcyan, CD27 APC-H7, CD45RO APC, HLA-DR FITC and CD38 PE (all from BD). Stained cells were finally fixed with 2% paraformaldehyde prior to acquisition. Acquisition was performed on a FACS CANTO II (BD). Compensation was conducted with antibody capture beads (BD) stained separately with the individual antibodies used in the test samples. Flow cytometry data was analyzed using FlowJo (version 9.5.3; Tree Star Inc.). Depending on the expression of CD27 and CD45RO markers on CD4 and CD8 T cells; T cell subsets were defined as follows: naïve (CD27^+^CD45RO^−^), “central-like” memory (CD27^+^CD45RO^+^), “effector-like” memory (CD27^−^CD45RO^+^) and “terminally differentiated” (CD27^−^CD45RO^−^) CD4 and CD8 T cells. In addition, total memory CD4 T cells were defined as the sum of central memory, effector memory and terminally differentiated CD4 T cells.

### Assessing CCR5 surface density on memory CD4 T cells

We used fresh, anti-coagulated whole blood in order to maximize CCR5 staining sensitivity and minimize staining variability that can arise due to cryopreservation of PBMC. The CCR5 surface density on total memory CD4 T cells was assessed using a strategy that rely on the absence of CCR5 on naïve CD4 T cells. We first standardized all CCR5 median fluorescence intensity (MFI) results, in order to compare CCR5 expression on memory CD4 T cells from different subjects and study visits. The CCR5 MFI value specific to CD45RO^+^ memory CD4 T cells was calculated and standardized by subtracting the CCR5-MFI on CD45RO^−^ naïve CD4 T cells from the same sample (ΔMFI). In addition, CCR5 MFI values specific to HLA-DR^+^ and HLA-DR^−^ memory CD4 T cells were calculated for each subject. All flow cytometric analyses were blinded for helminth and HIV infection status.

### Determination of plasma cytokines

Cryopreserved plasma samples from Trichuris infected subjects (n = 31) and randomly selected helminth-negative controls (n = 27) were tested in a single run to determine the concentration of the cytokines IFN-γ, TNFα, IL-1β, IL-2, IL-4, IL-5, IL-6, IL-9, IL-10, IL-12, IL-13, IL-17α and IL-22 at baseline using a multiplex cytometric bead array kit (eBioscience) as per manufacturer's instructions. Data acquisition was performed on a FACS Calibur (BD). The generated data was analyzed using FlowCytomix Pro 2.4 software (eBioscience).

### Statistical analysis

Data analyses were performed using Prism version 5.0 software (GraphPad, Inc.). Groups were compared using the Mann-Whitney test, paired observations (before and after helminth treatment) were compared using the Wilcoxon-matched pairs test and associations were determined by linear regression analysis, with p-values <0.05 regarded as significant. Figure and table legends describe which test was used in each case. Helminth specific analyses included all subjects with data who were infected with the respective helminth, meaning that subjects with multiple helminth infection were included in more than one of compared groups.

## Results

### Study population

Table 1 describes the characteristics of the WHIS study population. 246 adults HIV negative volunteers were included in the baseline analysis. 58.5% of these were female and the mean age was 33.6 years. 159 (64.6%) of 246 subjects were infected with a single helminth species and 57 (23.2%) had no helminth infection at baseline. The post treatment analysis excluded 69 subjects who were still (or again) helminth infected at the post treatment survey (n = 48) or who had no data for this survey (n = 21) resulting in 177 participants whose data were included in the post-treatment analysis.

### Systemic T cell activation in subjects infected with different helminth species

**Figure 1:**
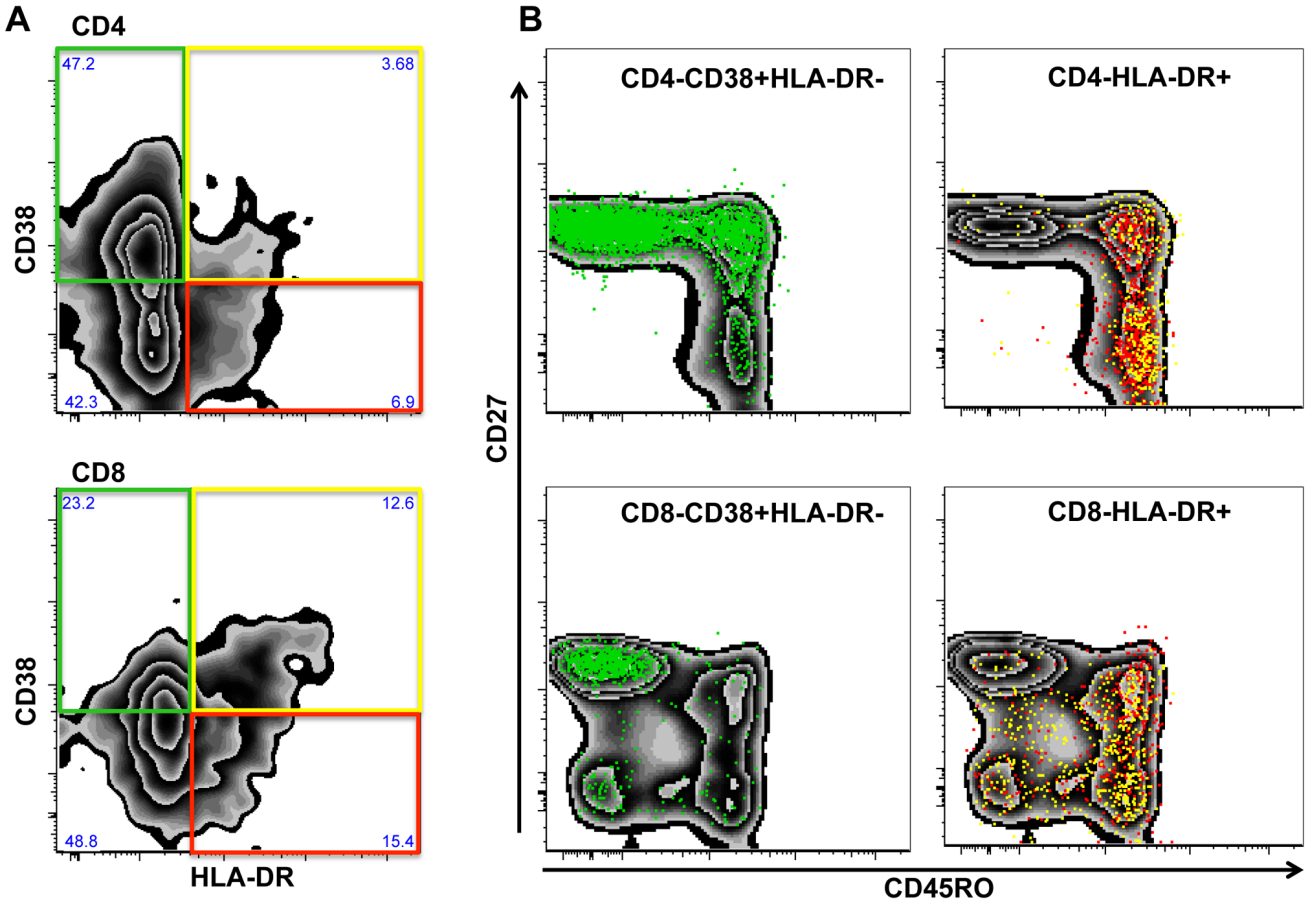
Expression of the T cell activation markers HLA-DR and CD38 on peripheral T cells. Shown in (A) is the gating strategy for flow cytometric analysis of CD38^+^HLA-DR^-^ (green), CD38^+^HLA-DR^+^ (yellow) and CD38^−^HLA-DR^+^ (red) CD4 (upper panel) or CD8 (lower panel) T cells of one representative subject. The distribution of CD4 (upper panels) and CD8 (lower panels) T cells expressing CD38^+^HLA-DR^−^ (left panels) or HLA-DR^+^ (right panels) is shown in (B) for CD45RO^−^CD27^+^ “naïve”, CD45RO^+^CD27^+^ “central memory like” and CD45RO^+^CD27^−^ “effector memory like” T cells.

To examine whether different helminth infections modulate systemic immune activation, we first studied the baseline expression of the T cell activation markers HLA-DR and CD38 on total CD4 and CD8 T cells in HIV negative volunteers with (n = 189) and without helminth infection (n = 57), as determined by the Kato-Katz method. At baseline, the vast majority (84%) of helminth infected individuals were infected with a single worm species as per Kato-Katz test performed during screening. Figure 1A shows a representative zebra plot and the gates that were used to study HLA-DR and CD38 expression on CD4 (upper panel) and CD8 T cells (lower panel). Generally HLA-DR expression was confined to the memory population of both CD4 and CD8 T cells, whereas CD38^+^/HLA DR^-^ CD4 and CD8 T cells consisted predominantly of CD45RO^-^CD27^+^ “naïve” T cells and a small proportion of CD27^+^ memory CD4 T cells (figure 1B, left panel). CD38 expression pattern on different memory CD8 T cell subsets had more inter-individual differences. Analysis of a subset of WHIS volunteers (n = 19) showed that HLA-DR^+^/CD38^+^ CD4 T cells were almost exclusively CD45RO^+^ memory T cells (median >90%) and a median of 63% co-expressed CD27 (supplementary figure S1), indicative of central memory like cells. Similarly, more than 90% of HLA-DR^+^/CD38^+^ CD8 T cells were memory T cells, but were distributed roughly equally between CD45RO^+^CD27^+^, CD45RO^+^CD27^−^ and CD45RO^−^CD27^−^ cell populations (supplementary figure S1).

Combined as one group, helminth infected subjects had only moderately and mostly insignificant increased frequencies of HLA-DR^+^ and/or CD38^+^ CD4 and CD8 T cells (Table 2) when compared to non-infected subjects. Nonetheless, in subjects with helminth infection the median proportion of HLA-DR^+^/CD38^+^ CD4 T cells was significantly elevated (2.16% versus 2.63%, p = 0.011) whereas median HLA-DR^+^/CD38^+^ CD8 T cell frequencies were moderately increased (5.50% versus 6.86%, p = 0.055). As expected, HIV^+^ subjects (n = 77) had highly elevated median frequencies of HLA-DR^+^/CD38^+^ CD8 T cells (25.5%) and CD4 T cells (14.3%) compared to all HIV^−^ subjects (data not shown), confirming the validity of our results. CD38^+^ CD4 and CD38^+^ CD8 T cell frequencies were also moderately but insignificantly increased (p<0.1 for both), although their predominantly “naïve” phenotype is counterintuitive for a T cell activation marker. Thus, taken together as a group, helminth infected individuals had significantly increased frequencies of “activated” HLA-DR^+^/CD38^+^ double-positive CD4, and a trend towards increased frequencies of HLA-DR^+^/CD38^+^ and CD38^+^ CD8 T cells.

**Table 2:** Expression of activation markers on CD4 and CD8 T cells in relation to chronic infection with different helminth species on HIV negative individuals.

	HIV negative		
	No helminth	All helminth+	P value*
N (%CD4)	51	197	
**%CD4+HLA-DR-CD38+**	42.0% (31.1–48.2%)	43.5% (34.6–53.3%)	0.1600
**%CD4+HLA-DR+CD38+**	2.16% (1.51–2.88%)	2.63% (1.90–3.66%)	**0.0108**
**%CD4+HLA-DR+CD38-**	4.51% (3.37–6.77%)	5.12% (3.60–7.07%)	0.2716
**%CD4+ Total HLA-DR+**	7.01% (5.07–9.78%)	7.80% (6.06–11.1%)	0.0892
**%CD4+ Total CD38+**	45.4% (34.2–50.7%)	46.4% (38.1–56.1%)	0.0878
**N (%CD8)**	52	195	
**%CD8+HLA-DR-CD38+**	24.2% (13.7–32.6%)	26.2% (15.2–38.2%)	0.1081
**%CD8+HLA-DR+CD38+**	5.50% (3.15–10.0%)	6.86% (4.14–12.9%)	0.0547
**%CD8+HLA-DR+CD38-**	11.1% (7.24–14.6%)	11.8% (6.84–20.3%)	0.4517
**%CD8+ Total HLA-DR+**	18.4% (12.5–25.0%)	21.4% (11.7–32.8%)	0.2681
**%CD8+ Total CD38+**	30.0% (21.8–40.8%)	34.9% (23.4–50.0%)	0.0617

*P values for comparison between helminth infected and non-infected controls were calculated using the Mann-Whitney test.

**Figure 2:**
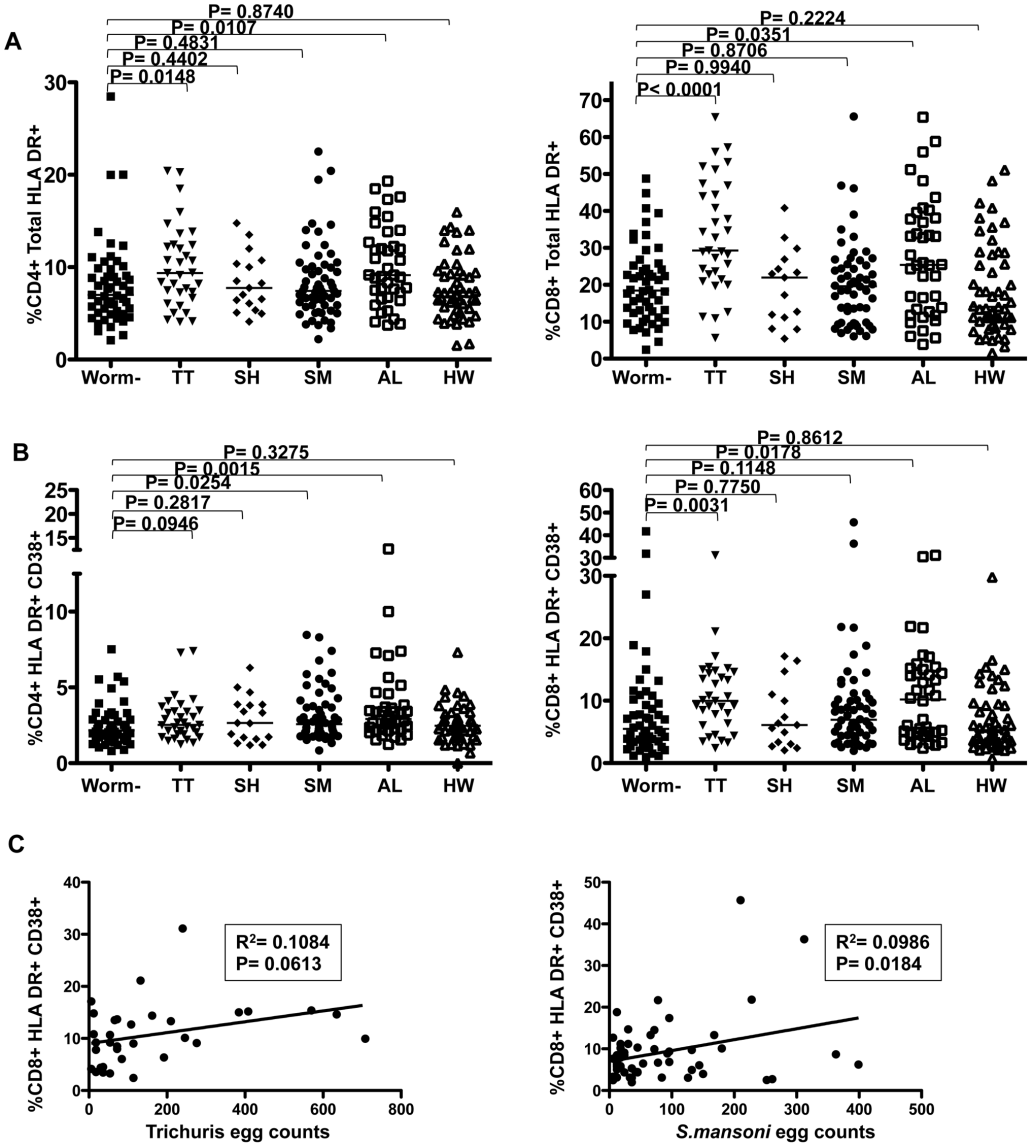
Expression of systemic T cell activation markers in relation to infection with different helminth species. The frequencies of HLA-DR^+^CD38^−^ and total HLA-DR^+^ (B) are shown on the y-axis for CD4 (left panels) and CD8 T cells (right panels). The worm infection status is indicated on the x-axis stratified into worm negative individuals or those infected with TT (*Trichuris trichiura*), SH (*Schistosoma haematobium*), SM (*Schistosoma mansoni*), AL (*Ascaris lumbricoides*) or HW (Hookworm). Statistical analysis was performed using Mann-Whitney test for comparing groups. Shown in (C) is a linear regression analysis between the frequency of HLA-DR^+^/CD38^+^ CD8 T cells and the worm egg counts (as measured by Kato-Katz method) within Trichuris (left panel) and *S. mansoni* (right panel) infected subjects.

We next compared these immune activation markers in HIV^−^ study volunteers after further stratification by helminth species: *Ascaris lumbricoides* (AL, n = 39), Hookworm (HW, n = 49), *Trichuris trichiura* (TT, n = 33), *Schistosoma mansoni* (SM, n = 59) and *Schistosoma haematobium* (SH, n = 17). We observed substantial differences in the expression of immune activation markers (HLA-DR and CD38) on T cells between different helminth infections. Particularly, subjects with TT and AL infection had significantly increased frequencies of activated T cells in the peripheral blood; In TT infected volunteers median frequencies of HLA-DR^+^ CD4 T cells (9.37% versus 7.01%, p = 0.015) and CD8 T cells (29.30% versus 18.44%, p<0.0001) were increased when compared to helminth negative subjects (figure 2A). Similarly, in AL infected subjects increased median frequencies of HLA-DR^+^ CD4 and CD8 T cells were also observed (%CD4, 9.14%, p = 0.011; %CD8, 25.4%, p = 0.035). SM or HW infections were not associated with substantial increases in HLA-DR^+^ CD4 T cell frequencies. To the contrary, there was a trend towards lower median frequencies of HLA DR^+^ CD8 T cells (14.01%) in HW infected volunteers compared to helminth negative subjects (p = 0.222). Median frequencies of HLA-DR^+^CD38^+^ CD4 T cells were significantly elevated in subjects infected with AL (1.3-fold, 2.92%, p = 0.002) and SM (1.2-fold (2.57%, p = 0.025), but not TT infections (1.3-fold, 2.52%, p = 0.095) when compared to non-infected individuals (2.16%, figure 2B left panel). Median frequencies of HLA-DR^+^CD38^+^ CD8 T cells were significantly elevated in subjects infected with TT or AL as compared to non-infected individuals (figure 2C right panel, 5.49% for none-infected, 9.96% (p = 0.003) for TT and 10.18% (p = 0.018) for AL). SM infected subjects had an insignificant increase in HLA-DR^+^CD38^+^ CD8 T cell frequencies (1.3-fold, 6.95%, p = 0.115).

Worm egg counts are an indicator of parasite burden within the infected host and we thus next wanted to assess whether increases in systemic T cell activation markers correlate with egg counts. Indeed, we observed a weak linear correlation between egg counts and the frequency of HLA-DR^+^CD38^+^ CD8 T cells in TT (p = 0.061, r^2^ = 0.11, figure 2C left panel) and SM (p = 0.018, r^2^ = 0.10, figure 2C right panel) infected individuals. In addition, for SM infected individuals, parasite egg counts weakly correlated with the frequency of HLA-DR^+^CD38^+^ CD4 T cells (p = 0.071, r^2^ = 0.05, supplementary figure S2). No linear relationship was observed for the frequency of HLA-DR^+^ T cells and parasite egg counts in subjects infected with neither TT nor SM. Interestingly we also did not find a significant linear relationship between egg counts and activated T cells in AL infected subjects (p>0.2, data not shown). In summary, these results show that TT, AL and SM infections are associated with systemic T cell activation. The weak linear correlation between egg counts and CD38^+^/HLA-DR^+^ T cells are consistent with a link between parasite burden and immune activation for TT and SM. Nonetheless, other factors might also contribute to immune activation in these individuals.

### Systemic T cell activation in Trichuris-infected subjects is linked to elevated levels of IL-1β and IL-10

Among the different helminth infections studied, TT infection was most significantly associated with increased systemic T cell activation. TT infection and T cell activation might also be linked to changes in systemic levels of pro-inflammatory cytokines, such as IL-1β, IL-6 or TNFα. We thus next measured plasma levels of 13 different cytokine in TT infected subjects (n = 31) and worm-negative controls (n = 27) simultaneously using a multiplex cytometric bead array for detection of pro-inflammatory cytokines (IL-1β, IL-6, TNFα, IL-17α, IFN-γ), “TH2” cytokines with anti-helminthic properties (IL-4, IL-5, IL-13) and the regulatory cytokine IL-10.

**Figure 3:**
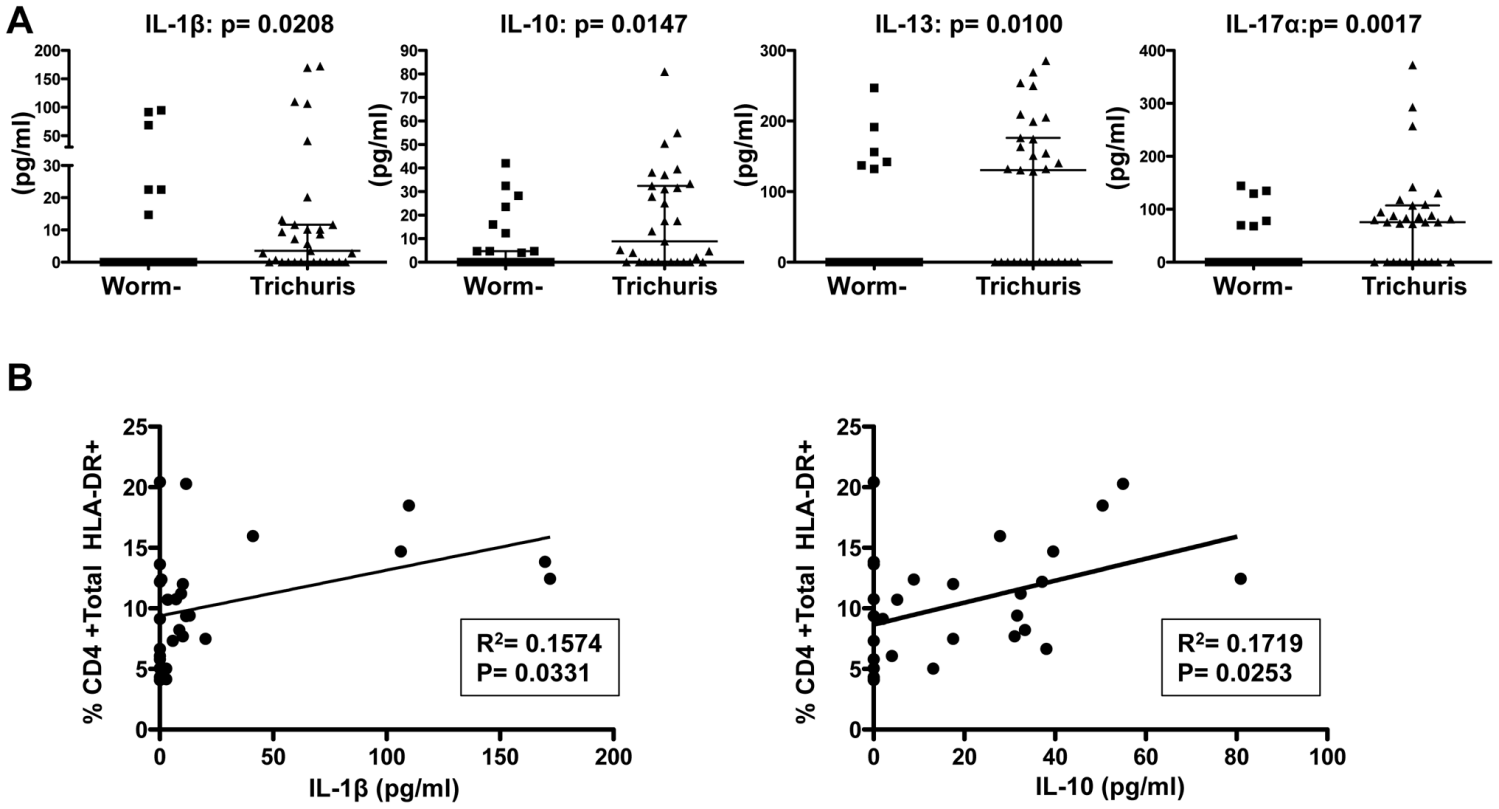
Elevation of pro-inflammatory and regulatory cytokine levels in the plasma of *Trichuris* infected subjects. Plasma levels of IL-1β, IL-10, IL-13 and IL-17α (y-axis) are shown in (A) for worm negative and Trichuris infected subjects. Shown in (B) is a linear regression analysis of the frequency of HLA-DR^+^ CD4 T cells and the plasma concentration of IL-1β (left panel) or IL-10 (right panel) within Trichuris infected subjects. Statistical analysis between groups was performed using the Mann-Whitney test for comparing groups.

TT infection was linked to increased levels of the pro-inflammatory IL-1β (median: 3.5 pg/ml versus 0.0 pg/ml, p = 0.021 figure 3A far left panel) and IL-17α (median: 75.4 versus 0.0 pg/ml, p = 0.002, figure 3A far right panel), but not to increased levels of IL-6 (median: 1.9 versus 2.0 pg/ml, p = 0.635, data not shown) or TNFα (median: 1.2 versus 2.8 pg/ml, p = 0.704, data not shown) when compared to worm-negative subjects. Furthermore, the majority of TT infected subjects also had elevated levels of IL-13 (median: 130.4 versus 0.0 pg/ml, p = 0.010, figure 3A right panel), but no detectable differences in IL-4 (median: 42.8 versus 25.5 pg/ml, p = 0.223, data not shown) or IL-5 (median: 0.0 versus 0.0 pg/ml, p = 0.289, data not shown). Interestingly, plasma levels of the regulatory cytokine IL-10 were also elevated in TT infected subjects (8.9 versus 0.0 pg/ml, p = 0.015, figure 3A left panel).

Within TT infected individuals, we next compared the plasma concentration of these cytokines with the frequency of HLA-DR^+^ T cells. Indeed, IL-1β plasma levels correlated positively with the frequency of HLA-DR^+^ CD4 (p = 0.033, r^2^ = 0.16, figure 3B left panel) and CD8 T cells (p = 0.014, r^2^ = 0.20, supplementary figure S3). Similarly, there was a strong correlation between IL-10 plasma levels and HLA-DR^+^ CD4 (p = 0.025, r^2^ = 0.17, figure 3B right panel) but not with HLA-DR^+^ CD8 T cells (p = 0.400, supplementary figure S3). IL-13 and IL-17 concentrations did not correlate with the frequency of HLA-DR^+^ CD4 or CD8 T cells (p>0.25). These data show that systemic activation of T cells is linked to the pro-inflammatory IL-1β and simultaneously to the regulatory IL-10.

Interestingly, plasma levels of IL-1β, IL-10, IL-17α and IL-13 closely correlated with each other and could only be detected in a subset of Trichuris infected individuals. For example, subjects with elevated IL-1β levels typically also had elevated IL-10 levels (p = 0.005, r^2^ = 0.24, supplementary figure S4), IL-13 (p<0.0001, r^2^ = 0.41, supplementary figure S4) and IL-17 (p<0.0001, r^2^ = 0.64, data not shown), suggesting that elevation of pro-inflammatory, anti-helminthic and regulatory cytokines in the plasma is closely linked in TT infected individuals.

### Surface density of HIV-coreceptor, CCR5 on peripheral memory CD4 T cells in relation to chronic infection with different helminth species

HIV transmission occurs almost exclusively with CCR5-tropic HIV strains [12] and CCR5-tropic strains also predominate in the majority of individuals during chronic infection [13]. The expression of CCR5 on activated CD4 T cells is likely to contribute to the early selection of CCR5-tropic strains [14]. CCR5 expression is common on memory CD4 T cells in mucosal lymphoid tissues, the mucosa of the reproductive tract and intestine, the lungs and inflamed tissues [15]–[17] (also reviewed in [18]).

**Figure 4:**
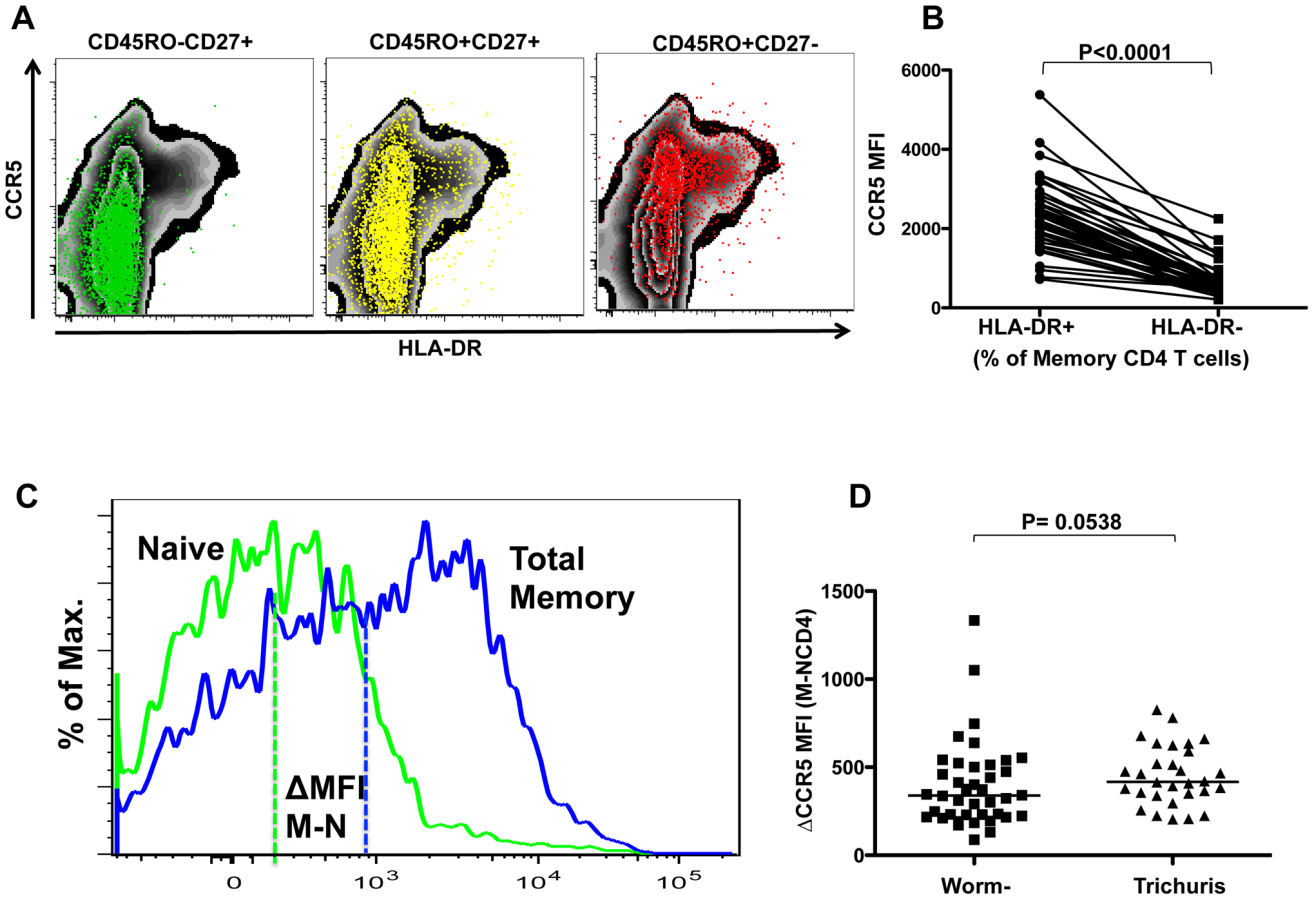
HIV co-receptor expression on peripheral memory CD4 T cells in relation to Helminth infection. (A) Shows a representative zebra overlay dot plot of CCR5 and HLA-DR expression on CD4 T cells. CD45RO^−^CD27^+^ “naïve” (green, left panel), CD45RO^+^CD27^+^ “central memory like” (yellow, middle panel) and CD45RO^+^CD27^−^ “effector memory like” (red, right panel) CD4 T cell subsets are indicated. Comparison of CCR5 Median Fluorescent Intensities (MFI) between HLA-DR^+^ and HLA-DR^−^ memory CD4 T cells in HIV negative, Worm negative subjects is shown in (B). Shown in (C) is a representative histogram overlay for CCR5 staining on total memory CD4 T cells (blue) and naïve CD4 T cells (green) including the subset specific MFI. The method of calculation of the CCR5 ΔMFI is indicated in the diagram. Comparison of ΔMFI between Worm negative and Trichuris is shown in (D). Statistical analyses were performed using Mann-Whitney test for comparing groups and Wilcoxon-matched pairs test for comparing paired observations.

Generally, CCR5 expression was largely absent from CD45RO^−^ CD27^+^ (naïve) CD4 T cells, whereas less mature CD45RO^+^CD27^+^ memory CD4 T cells included substantial proportion of CCR5^+^ cells (typically 30–50%) with a small proportion co-expressing HLA-DR. A representative zebra plot overlay of these T cell subsets delineated by CD27 and CD45RO expression is shown in figure 4A. More mature CD45RO^+^CD27^−^ memory CD4 T cells contained the largest fraction of CCR5^+^ cells (typically 50–80%) and also HLA-DR^+^ memory CD4 T cells frequently co-expressed CCR5. In fact, a higher median density of CCR5 was detected on activated memory (HLA-DR^+^) CD4 T cells in all studied groups than in non-activated memory (HLA-DR^−^) CD4 T cells (all: p<0.0001, data not shown). For example, the CCR5 median density on HLA-DR^+^ memory CD4 T cells was more than 3-fold increased compared to HLA-DR^−^ memory CD4 T cells in HIV negative, none-helminth infected subjects (Medians: 2198 versus 638 respectively, p<0.0001, figure 4B).

In the present study, we wanted to address the question whether systemic immune activation during chronic infection with different helminth species might also be linked to an increase of CCR5 surface expression on the memory CD4 T cells. In order to compare CCR5 expression density on total memory CD4 T cells from different subjects and study visits, we first determined the CCR5 MFI on CD45RO^+^ memory and CD45RO^−^ naïve CD4 T cells and standardized CCR5 MFI results for CD45RO^+^ memory CD4 T cell subset by subtracting CCR5 MFI for CD45RO^−^ naïve CD4 T cells for each sample (Figure 4C). In addition, the frequency of CCR5 expression on activated (defined by the expression of HLA-DR) total memory CD4 T cells was studied.

None of the helminth infections was associated with substantial changes in the expression of CCR5 on memory CD4 T cells. TT infection was however associated with a moderate but insignificant increase of the ΔCCR5 MFI (memory-naïve) as compared to the worm-negative control group (1.2-fold, medians: 417 versus 339 respectively, p = 0.054, Figure 4D). Furthermore, we also observed a trend towards a moderate increased frequencies of CCR5^+^/HLA-DR^+^ double positive memory CD4 T cells in the AL infected individuals (median: 7.24%) compared to the control group (median: 5.70%, p = 0.093, data not shown), even though no change in ΔCCR5 MFI could be observed in this group when compared to controls (median: 381, p =  0.542, data not shown). No significant change in the frequencies of CCR5^+^/HLA-DR^+^ memory CD4 T cells could be observed in TT infected group (median: 6.60%) when compared to the control group (median: 5.70% p = 0.204, data not shown). These results suggest that TT infection is associated with a moderately higher density of CCR5 on circulating memory CD4 T cell whereas AL infection is linked to a moderate increase in frequencies of activated CD4 T cells that co-express CCR5.

### Effect of helminth treatment on systemic T cell activation and CCR5 surface density on memory CD4 T cells

Whether treatment of helminth infections reduces systemic immune activation in HIV negative individuals has not been explored so far. We only included subjects with no detectable helminth infection post-treatment (n = 177) into this analysis. We first studied the effect of one dose of Albendazole/Praziquantel treatment on eosinophil counts to determine whether helminth treatment has an effect on helminth-induced eosinophilia (Table 3). At baseline, helminth infection was associated with eosinophilia (p = 0.004, p-value not shown in Table 3). More specifically, eosinophiles were highest during infections with TT (median: 400/μl, p = 0.009) followed by infections with AL (median 280/μl, p = 0.023), SM (median: 275/μl, p = 0.004) and HW (median: 220/μl, p = 0.033, p-values not shown in Table 3). 3 months post treatment eosinophil counts decreased in subjects infected with HW (p = 0.003), SM (p = 0.001) and AL (p = 0.115). Only TT infected subjects remained with very high eosinophil counts after treatment (median: 300/μl vs. 400/μl, p = 0.456). Compared to worm negative control subjects, who showed no effect of worm treatment (p = 0.416), the median change in eosinophil counts post treatment differed significantly for SM (p = 0.036) infected subjects.

**Table 3:** Expression of activation markers on CD4 and CD8 T cells of HIV negative individuals before and after de-worming (n = 177**).

	Worm-	TT+	SH+	SM+	AL+	HW+	All worms+
**Eosinophiles (cells/μl)**							
**N**	30	19	11	32	22	28	96
Median at baseline	165	400	200	275	280	220	260
Median 3 m*	120	300	160	225	220	150	175
p (Baseline vs 3 m)°	0.4159	0.4563	0.4227	**0.0007**	0.1150	**0.0031**	**0.0001**
p (infected vs controls)♦		0.6813	0.5362	**0.0357**	0.2283	0.0650	0.0965
**HLA-DR+CD4 T cells (%)**							
**N**	33	19	10	45	22	37	115
Median at baseline	7.81	10.71	7.35	7.00	9.04	7.24	7.40
Median 3 m*	7.63	7.77	7.60	7.21	8.26	7.47	7.40
p (Baseline vs 3 m)°	0.3391	0.0990	0.3329	0.2041	0.3464	0.8386	0.9366
p (infected vs controls)♦		0.2830	0.5085	0.1411	0.6613	0.5843	0.5160
**HLA-DR+CD8 T cells (%)**							
**N**	33	19	9	42	23	38	112
Median at baseline	18.62	32.76	17.21	19.03	24.30	13.81	19.03
Median 3 m*	19.43	21.59	20.49	14.84	22.93	16.33	18.45
p (Baseline vs 3 m)°	0.1266	**0.0033**	0.8590	0.6707	**0.0106**	0.5473	0.1919
p (infected vs controls)♦		0.1358	0.5707	0.6159	0.0909	0.1528	0.7236
**ΔCCR5 MFI on CD4**							
**N**	26	17	9	24	13	19	71
Median at baseline	343.0	420.0	434.0	289.5	390.0	274.0	351.0
Median 3 m*	400.5	282.0	234.0	251.5	232.0	178.0	232.0
p (Baseline vs 3 m)°	0.5338	**0.0031**	0.3743	0.4073	**0.0030**	0.2954	**0.0013**
p (infected vs controls)♦		**0.0416**	0.7059	0.8384	**0.0264**	0.7217	0.2458

*median values 1–3 months after helminth treatment.

° P values for difference between baseline and 1–3 months follow up median values performed using the Wilcoxon-matched pairs test.

♦ P values for median change after treatment between helminth infected and non-infected controls were performed using the Mann-Whitney test.

**Please note that not all 177 subjects had data for each of the examined parameters, and that subjects with multiple helminth infections were included more than once for each of the helminths that they were infected with. Thus the number of subjects for each helminth infection group does not add up to 177.

Next, we studied the effect of helminth treatment on T cell activation markers. Importantly, flow cytometric analysis of T cell activation markers and CCR5 expression was blinded to helminth infection status at baseline. We compared the frequencies of HLA-DR^+^ and HLA-DR^+^/CD38^+^ on CD4 and CD8 T cells and in addition studied the CCR5 expression density on CD4 T cells at 1–3 months (Table 3) in subjects with and without helminth infection at baseline. Surprisingly, only very minor changes in HLA-DR expression on CD4 T cells could be detected with no substantial differences between helminth infected subjects and the control group. The largest difference between the pre- and post-treatment visit was detected for TT infected subjects from a median of 10.71% HLA-DR^+^ CD4 T cells to a median of 7.77% (p = 0.099), but even this change did not differ significantly to that in the control group (p = 0.283). Median frequencies of HLA-DR^+^ CD8 T cells decreased substantially in TT (32.76% to 21.59%, p = 0.003) and AL (24.30% to 22.93%, p = 0.011) infected individuals, whereas it slightly insignificantly increased in HW infected individuals. We also observed a very minor and insignificant increase in HLA-DR^+^ CD8 T cell frequencies in the control group (18.62% to 19.43%, p = 0.127). Compared to the control group, the decrease in HLA-DR expression was more pronounced but still insignificant in TT (p = 0.136) and AL (p = 0.091) infected subjects.

Changes in HLA-DR^+^/CD38^+^ CD8 T cells (data not shown) were similar to HLA-DR^+^ CD8 T cells and the biggest declines were observed for TT (median: 9.02% to 6.61%, p = 0.008) and AL (median: 9.96 to 6.83%, p = 0.128), whereas median frequencies in the control group only declined from 6.03% to 5.52% (p = 0.161). However differences in HLA-DR expression dynamics between any of the worm infected groups and the control group were insignificant.

We next analyzed the effect of helminth treatment on CCR5 density on the cell surface of memory CD4 T cells (ΔCCR5 MFI, Table 3). A significant decline in CCR5 density was observed in subjects treated for TT (median: 420 to 282, p = 0.003) and AL (median: 390 to 232, p = 0.003), whereas no significant decline was observed in helminth negatives (median: 343 to 400.5, p = 0.534) and in SM infected subjects (median 289.5 to 251.5, p = 0.407). Compared to the control group, the treatment induced change in CCR5 density on memory CD4 T cells was significant in TT and AL infected subjects (p = 0.041 and 0.026 respectively).

## Discussion

It has been hypothesized that systemic immune activation caused by chronic helminth infection contributes to increased HIV transmission in sub-Saharan Africa [1] and therefore to the high HIV prevalence in this region. This hypothesis is supported by observations that low systemic T cell activation is linked to HIV resistance in highly exposed HIV uninfected individuals [8]–[10]. Furthermore, it is well established that T cell activation and proliferation facilitate efficient HIV replication in vivo and in vitro [18]–[20]. Previous studies support the concept that helminth infections are associated with systemic T cell activation [3]–[5]. However, whether helminths are a primary cause of systemic T cell activation in populations from endemic areas of Africa is not entirely clear, because these studies did not specifically investigate immune activation before and after helminth treatment, nor did they differentiate between different helminth species. To fill this gap, we studied systemic T cell activation and HIV co-receptor expression in relation to helminth infection within the large WHIS cohort from Mbeya region, Tanzania, before and after deworming with Albendazole and Praziquantel.

Our results show that Trichuris, but also Ascaris and *S. mansoni* infections are linked to increased frequencies of “activated” CD4 and/or CD8 T cells defined by expression of HLA-DR alone or in combination with CD38. Of note, increased T cell activation was quite dramatic for CD8 T cells during Trichuris infection, whereas Ascaris infection was rather associated with more activated CD4 T cells. It should nonetheless be noted that frequencies of activated T cells varied greatly between individuals infected with Ascaris or Trichuris, suggesting that causes of systemic T cell activation are multifactorial. Other factors such as additional persistent infections (as observed during HIV infection) or host genetic differences are likely to also influence T cell activation status. Hookworm infection was associated with a moderate, but insignificant decrease in the frequency of HLA-DR^+^ CD8 T cells. Thus, while these results partially agree with previously published data that helminth infections are associated with T cell activation [3], [4], our results suggest that not all helminth species are necessarily associated with systemic T cell activation and that Hookworms might even have an opposing effect.

Independent of helminth infection status, CD38 expression alone was a characteristic of “naïve” CD27^−^/CD45RO^−^ CD4 and CD8 T cells, whereas co-expression with HLA-DR was exclusively detected on memory T cells. In our study population it is thus unlikely that CD38 expression on naïve T cells is a marker of T cell activation and we therefore concentrated on HLA-DR expression alone or in combination with CD38.

The etiology of helminth-associated T cell activation is not known. Trichuris and *S. mansoni* egg counts are positively correlated with the frequency of HLA-DR^+^/CD38^+^ CD8 and CD4 T cells (figures 2D and S2), respectively, suggesting that high parasite burdens contribute to systemic T cell activation. Moreover, Trichuris infection was associated with increased plasma levels of pro-inflammatory (IL-1β and IL-17α), anti-helminthic (IL-13) and regulatory (IL-10) cytokines, which closely correlated with each other; showing a mixed cytokine response to infection with Trichuris. Faulkner et al. also observed a similar mixed cytokine response in the blood of Cameroonian children with Trichuris and Ascaris infections following an exposure to Trichuris antigens [21]. Of interest, IL-1β and IL-10 concentrations in our Trichuris infected volunteers positively correlated with the frequency of HLA-DR^+^ CD4 and/or CD8 T cells, linking systemic T cell activation to the pro-inflammatory IL-1β and simultaneously to the regulatory IL-10. It is therefore possible that the immune response to Trichuris infection causes immune activation through the induction of pro-inflammatory cytokines, but also evokes a systemic regulatory and anti-helminthic cytokine response. Our data thus confirm previous reports that Trichuris infections are associated with increased IL-10 levels [21], [22] and provide a possible link between helminth associated systemic immune activation, hypo responsiveness and anergy [23], [24].

The differences in T cell activation profile between the different helminth species is intriguing and surprising, particularly for Hookworm versus Trichuris infection. Both species interact closely with the gut epithelium, but only Hookworms feed on blood and thus are probably more exposed to circulating immune cells than Trichuris. Thus it is counterintuitive that Trichuris, but not Hookworms are associated with increased levels of activated, HLA-DR^+^ T cells. Gaze et al. have demonstrated that experimental human Hookworm infection induces a systemic Hookworm-specific cellular immune responses, which is characterized by production of several TH2, TH1 and the regulatory cytokines upon re-stimulation of PBMC in vitro [25], suggesting that Hookworm infection is immunogenic. One potential difference between the two species could be associated plasma levels of the pleiotropic IL-17. We found increased plasma levels of IL-17α in association with Trichuris infection, whereas George et al. have found decreased levels of this cytokine in Hookworm infected individuals [26]. IL-17 induces IL-1β production in human Macrophages [27] and our results show a close correlation between IL-17α and IL-1β plasma levels. Furthermore IL-1β levels correlated with HLA-DR expression on circulating CD4 T cells in Trichuris infected individuals. Thus, while remaining speculative, differences in the induction of the IL-17 pathway might play a role in the observed difference in systemic T cell activation between Hookworm and Trichuris infection.

Very high frequencies of HLA-DR^+^ (and CD38^+^) “activated” T cells occur also during HIV infection [28]–[34] and were characteristic for HIV^+^ WHIS study participants as well (unpublished data). It has previously been suggested that translocation of immunostimulatory microbial compounds, such as Lipopolysaccharide (LPS) contribute to systemic immune activation during HIV infection [35], [36]. Due to the close interaction of Trichuris with the intestinal epithelium we hypothesized that immune activation during Trichuris infection might be caused by microbial translocation and therefore studied LPS levels in subjects with and without Trichuris infection. However, we did not detect increased LPS levels in Trichuris infected subjects (data not shown). Furthermore, in vitro stimulation of PBMCs for 48 h with LPS did not induce an “activated” T cell phenotype, whereas stimulation with the T cell growth factor IL-15 did (data not shown), arguing against this hypothesis. In addition, experimental (non-productive) Trichuris infection of Rhesus macaques with inflammatory bowel disease (IBD) actually decreases markers (sCD14) of microbial translocation and IBD associated T cell proliferation [37], further arguing against the notion that microbial translocation is a cause of systemic immune activation in Trichuris infected individuals. Other groups have detected increased plasma levels of LPS in association with *S. mansoni* and Hookworm infection [26], [38]. *S. mansoni* infection indeed correlated with increased levels of HLA-DR^+^/CD38^+^ CD4 and CD8 T cells. However, Hookworm infection was not associated with increased, but rather with slightly lower frequencies of “activated” HLA-DR^+^ CD8 T cells. Thus, while the etiology of T cell activation during helminth infection and its connection to microbial translocation remains to be fully elucidated, it is important to note that despite its reported association with microbial translocation [26], Hookworm infection was rather linked with a trend to lower frequencies of HLA-DR^+^, “activated” CD8 T cells.

To determine whether helminth-associated systemic immune activation was primarily caused by helminth infections, we studied the effect of one dose of Albendazole/Praziquantel treatment on reducing systemic immune activation. It is well established that infections with helminths are associated with eosinophilia (reviewed in [39], [40]). Eosinophils decreased 3 months post treatment in subjects infected with Hookworm, *S. mansoni* and to a lesser degree Ascaris, but remained exceptionally high in subjects infected with Trichuris, demonstrating a strong effect of Albendazole/Praziquantel treatment on helminth-induced immune system modulation with the exception of Trichuris infections. Having observed this, we studied modulation of activated T cells frequencies post-treatment. HLA-DR^+^ T cell frequencies most profoundly dropped in subjects infected with Trichuris and Ascaris but increased in those infected with Hookworm, which is consistent with our observations at baseline. Nonetheless, the changes were insignificant when directly compared to the helminth negative control subjects, who were also treated. The relatively minor effect of helminth-treatment in Trichuris infected volunteers on T cell activation and eosinophilia might be explained by the fact that Albendazole treatment might not have completely cleared Trichuris infection. Indeed, it is well known that Abendazole is not fully effective for treating Trichuris infection [41]. Supporting this argument, 30% (9 of 30) Trichuris infected subjects (which were excluded in the post-treatment analysis) had detectable Trichuris eggs post-treatment as per Kato-Katz test and a more sensitive test probably would have detected even more infections. A recent study has demonstrated only 10% cure rate using an identical Albendazole treatment as used during the WHIS study [42]. More effective treatment options [42] could help to clarify the effect of Trichuris eradication on systemic immune activation. However, based on our data, we cannot exclude the possibility that other environmental factors associated with the presence of Ascaris or Trichuris worms also contributed to increased systemic T cell activation in WHIS study volunteers.

To our knowledge, only one other longitudinal study has studied the effect of worm treatment on reduction of T-cell activation in HIV negative individuals [43]. Kassu et al. observed no significant changes in the expression of HLA-DR and CD38 on CD4 T cells in HIV negative subjects six months after helminth treatment but a significant decline in frequencies and numbers of HLA-DR^+^/CD38^+^ CD8 T cells. This study however did not distinguish between helminth and other intestinal parasites and was limited by a small sample size. Our study therefore provides for the first time extensive evidence on helminth associated systemic T cell activation and the impact of Albendazole/Praziquantel treatment.

Is it possible that these activated T cells are helminth-specific? After Yellow fever (YF) vaccination co-expression of HLA-DR and CD38 is characteristic for recently activated, proliferating (Ki67^+^) YF-specific CD8 T cells during the peak response [44] and thus this is one possible explanation. However, it is counterintuitive that during Trichuris infection such large fractions of CD8 T cells participate in the anti-helminthic immune responses.

Is HLA-DR expression a marker of cycling T cells? It has been documented that HIV associated immune activation defined by HLA DR alone or in combination with CD38 is linked to substantial increases in T cell proliferation [45]. HLA-DR expression on CD25^+^CD127^-^ CD4 T cells correlate with T cell proliferation during HIV infection [46] and as mentioned above HLA-DR and CD38 is characteristic for recently activated, proliferating (Ki67^+^) YF-specific CD8 T cells after YF vaccination. Based on these previous findings, we propose that increased frequencies of HLA-DR expressing T cells are a marker of increased systemic T cell proliferation in helminth infected subjects.

Although a trend towards increased CCR5 density on memory CD4 T cells and an increased frequency of CCR5^+^/HLA-DR^+^ memory CD4 T cells was observed in Trichuris and Ascaris infections respectively, which is in line with previous reports [4], [7], these values varied greatly between different individuals, prohibiting conclusions on modulation of cellular susceptibility to HIV infection caused by these helminth species [12], [47]–[49]. However our data clearly shows that independent of helminth infection, activated HLA-DR^+^ CD4 T cells express very high levels of CCR5 on their surface potentially facilitating cell entry of HIV.

In conclusion, not all studied helminth species modulated the systemic immune system in the same manner. Particularly, Trichuris, Ascaris and *S. mansoni* infections correlate with increased expression of T cell activation markers with relatively little effect of helminth treatment compared to helminth-negative controls. Contrary, Hookworm infection was associated with slightly decreased frequency of HLA-DR expressing CD8 T cells. Although we fail to demonstrate a strong effect of helminth treatment on T cell activation, the link between parasite burden and activated T cells during Schistosome and Trichuris infection suggest a causal link between the infection and immunomodulation. Because systemic T cell activation potentially contributes to increased HIV transmission risk [8]–[10] through facilitation of early systemic dissemination of the virus, our data support the concept that helminth infections, which are linked to systemic Immune activation and potentially increase CCR5 density on memory CD4 T cells, such as Trichuris infection, could indeed also contribute to increased HIV transmission risk during sexual activity.

## Supporting Information

Figure S1 **Frequency of activated cells within different T cell subsets.** Shows the frequency of CD45RO^-^CD27^+^ “naïve”, CD45RO^+^CD27^+^ “central memory like”, CD45RO^+^CD27^−^ “effector memory like” and CD45RO^−^CD27^−^ “terminally differentiated” T cells that also co-express CD38^+^HLA-DR^+^ on CD4 (left panel) and CD8 (right panel) T cells.
(TIF)

Figure S2 **Frequency of activated CD4 T cells is linked to** ***S. mansoni*** **egg count.** Linear regression analysis between the frequency of HLA-DR^+^/CD38^+^ CD4 T cells and the worm egg counts (as measured by Kato-Katz method) within *S. mansoni* infected subjects is shown.
(TIF)

Figure S3 **Frequency of systemic activated CD8 T cells in Trichuris-infected subjects is linked to elevated levels of IL-1β and IL-10.** Linear regression analysis between the frequency of HLA-DR expression on CD8 T cells and the plasma concentration of IL-1β (left upper panel) or IL-10 (left lower panel) within Trichuris infected subjects is shown. Shown in the right upper panel is the linear regression analysis between the frequency of HLA-DR^+^/CD38^+^ CD8 T cells and the plasma concentration of IL-1β.
(TIF)

Figure S4 **Linear correlation between plasma levels of IL1β and IL-10 within** ***Trichuris*** **infected subjects.** Linear regression analysis between the plasma levels of IL-1β and IL-10 (upper panels) or IL-13 (lower panels) is shown. Cytokine concentration in the plasma was measured as pg/ml (left panel) or Median Fluorescent intensity (right panel).
(TIF)
