# Supplementary figures and images for: Helminth-Associated Systemic Immune Activation and HIV Co-receptor Expression: Response to Albendazole/Praziquantel Treatment
Source: PLoS Negl Trop Dis. 2014 Mar 27;8(3):e2755. doi: 10.1371/journal.pntd.0002755 (PMC3967945; doi:10.1371/journal.pntd.0002755)

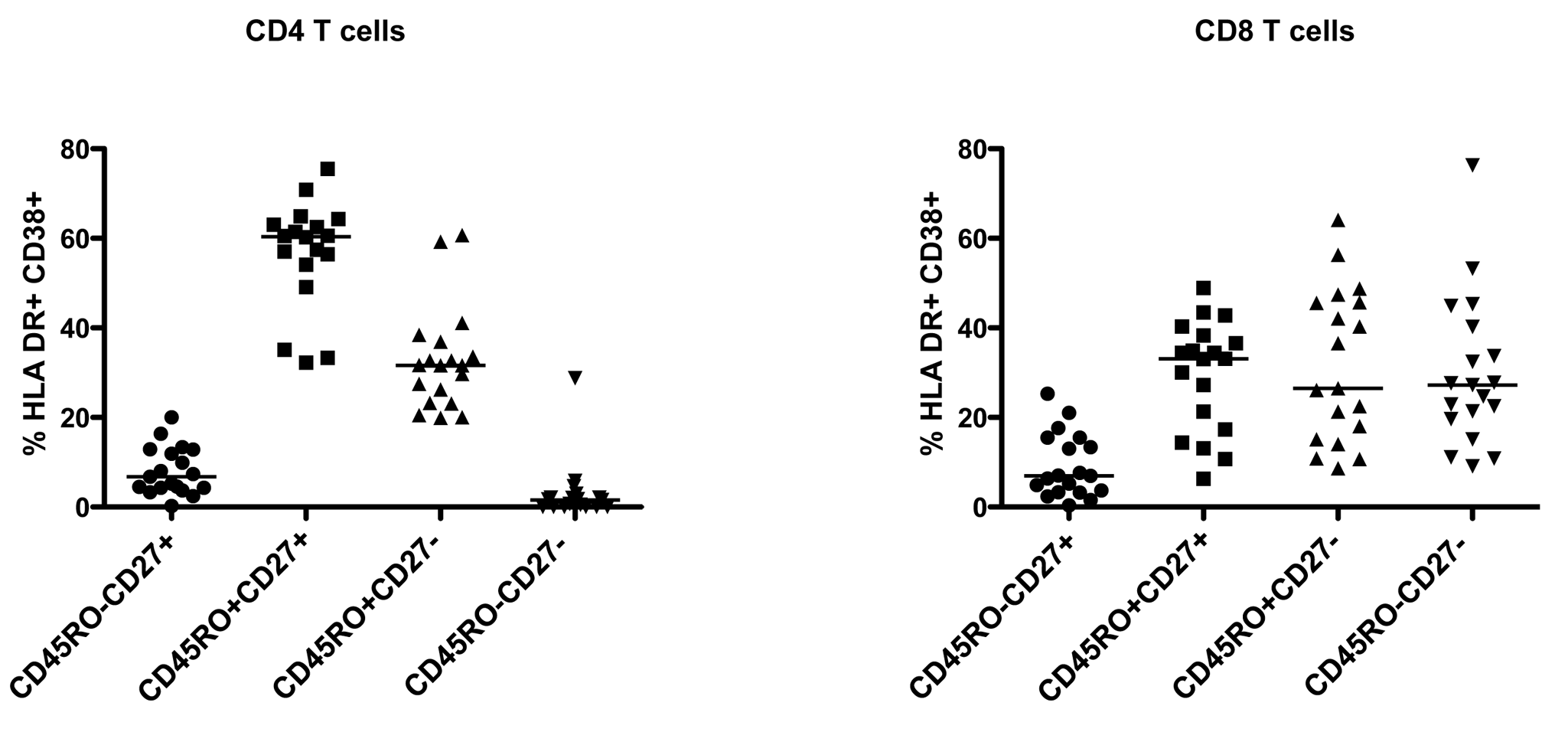

Supplement: Figure S1 — Frequency of activated cells within different T cell subsets. Shows the frequency of CD45RO-CD27+ “naïve”, CD45RO+CD27+ “central memory like”, CD45RO+CD27− “effector memory like” and CD45RO−CD27− “terminally differentiated” T cells that also co-express CD38+HLA-DR+ on CD4 (left panel) and CD8 (right panel) T cells. (TIF) [file pntd.0002755.s001.tif]

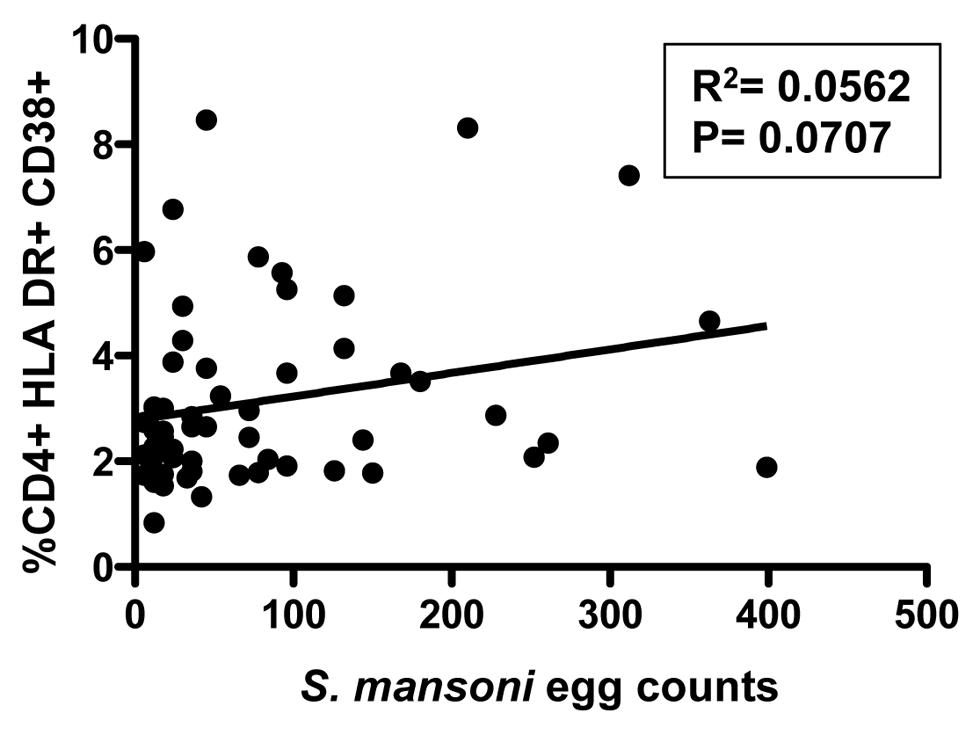

Supplement: Figure S2 — Frequency of activated CD4 T cells is linked to S. mansoni egg count. Linear regression analysis between the frequency of HLA-DR+/CD38+ CD4 T cells and the worm egg counts (as measured by Kato-Katz method) within S. mansoni infected subjects is shown. (TIF) [file pntd.0002755.s002.tif]

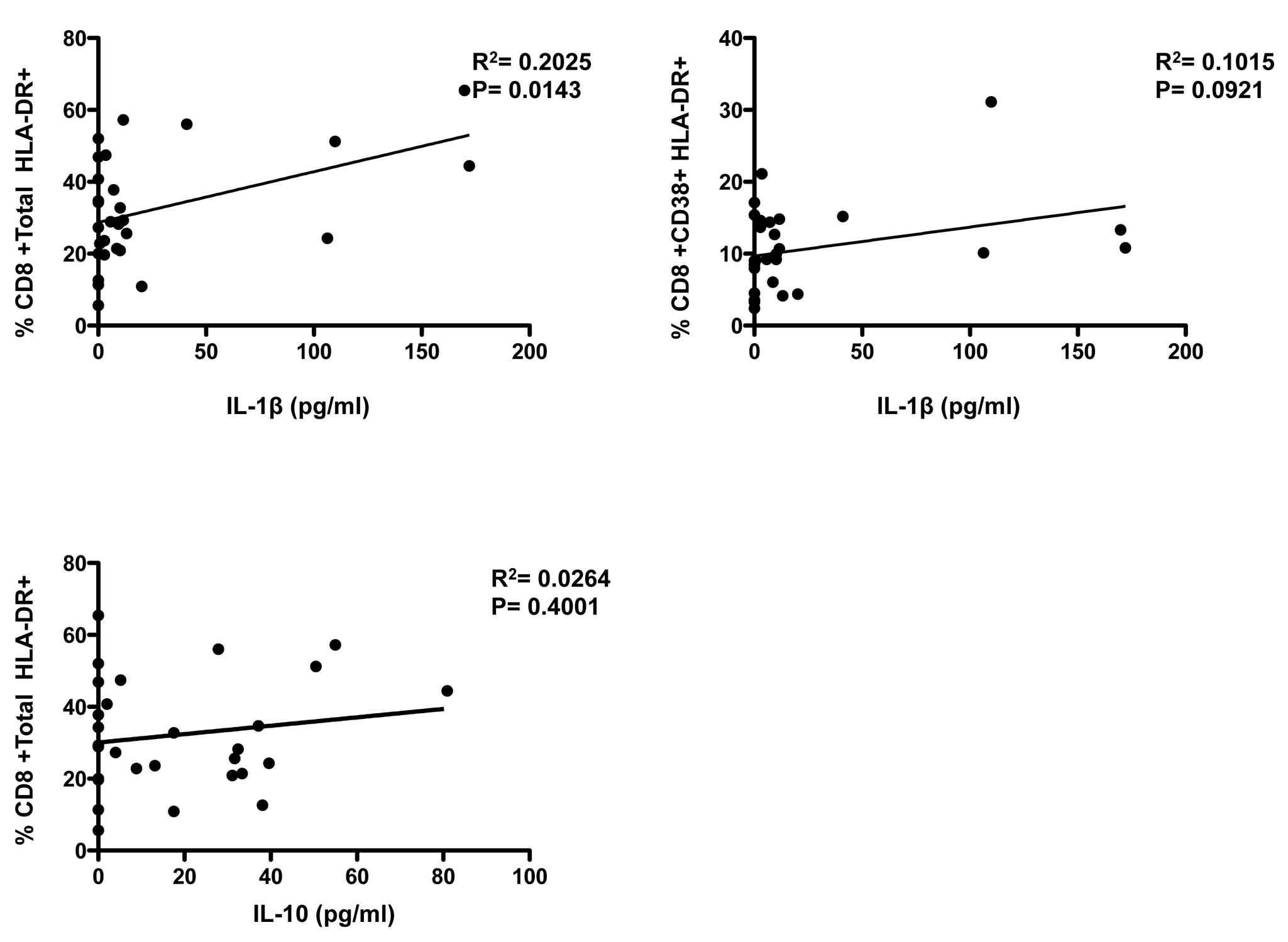

Supplement: Figure S3 — Frequency of systemic activated CD8 T cells in Trichuris-infected subjects is linked to elevated levels of IL-1β and IL-10. Linear regression analysis between the frequency of HLA-DR expression on CD8 T cells and the plasma concentration of IL-1β (left upper panel) or IL-10 (left lower panel) within Trichuris infected subjects is shown. Shown in the right upper panel is the linear regression analysis between the frequency of HLA-DR+/CD38+ CD8 T cells and the plasma concentration of IL-1β. (TIF) [file pntd.0002755.s003.tif]

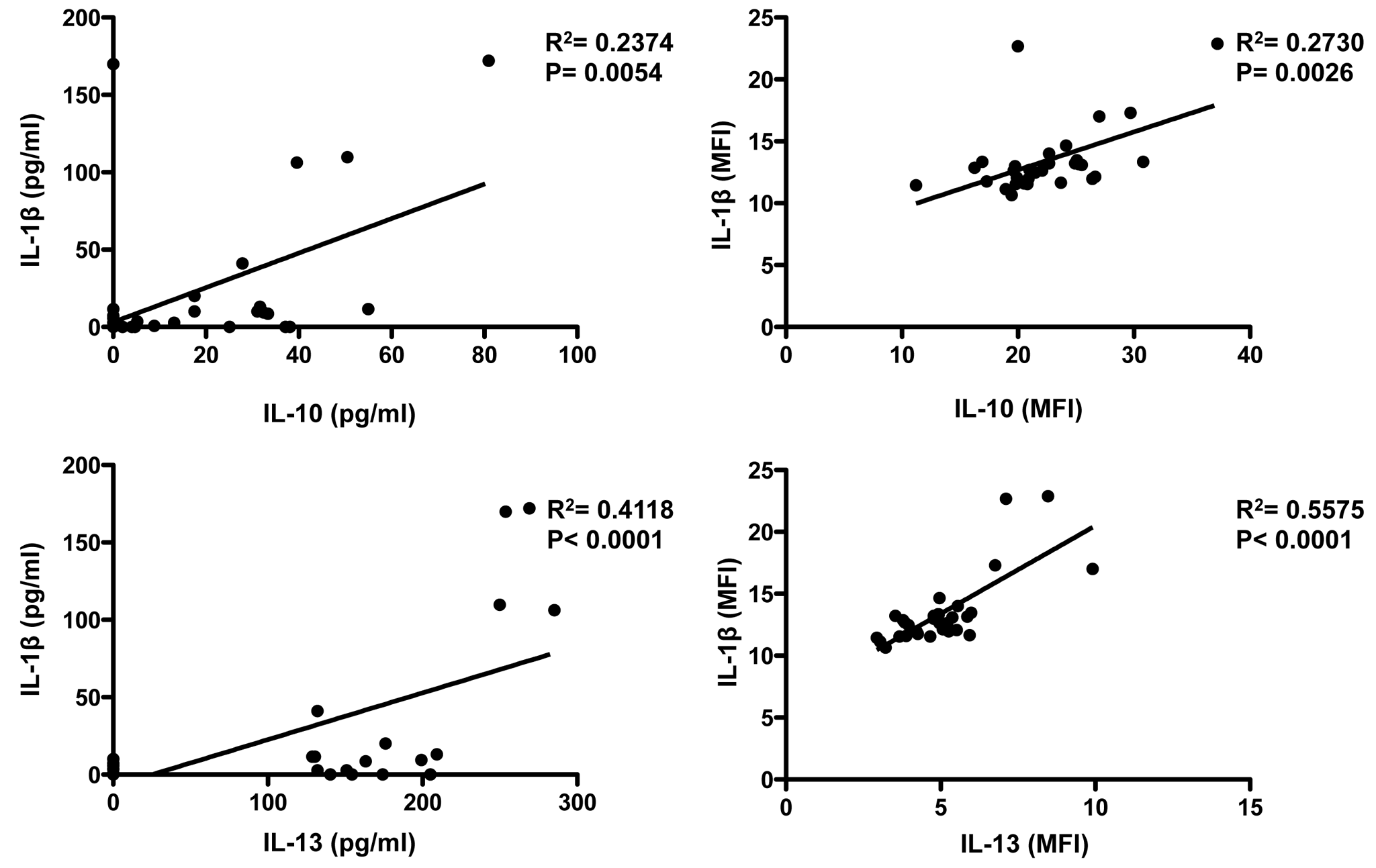

Supplement: Figure S4 — Linear correlation between plasma levels of IL1β and IL-10 within Trichuris infected subjects. Linear regression analysis between the plasma levels of IL-1β and IL-10 (upper panels) or IL-13 (lower panels) is shown. Cytokine concentration in the plasma was measured as pg/ml (left panel) or Median Fluorescent intensity (right panel). (TIF) [file pntd.0002755.s004.tif]
